# Association of apolipoprotein E (*ApoE*) polymorphisms with risk of primary hyperuricemia in Uygur men, Xinjiang, China

**DOI:** 10.1186/s12944-015-0025-2

**Published:** 2015-04-12

**Authors:** Yu-Ping Sun, Bei Zhang, Lei Miao, Xian-Min Wang, Jia-Hui Yu, Li Luo, Lu Ying, Gao Xin, Gulinizha Haliakpaer, He Xia, Hua Yao

**Affiliations:** College of Basic Science, Xinjiang Medical University, Urumqi, Xinjiang China; School of Public Health, Xinjiang Medical University, Urumqi, Xinjiang China; The Fourth Affiliated Hospital, Xinjiang Medical University, Urumqi, Xinjiang China; The Key Laboratory of Metabolic Diseases, Department of Education, Xinjiang Uygur Autonomous Region, The First Affiliated Hospital, Xinjiang Medical University, Urumqi, Xinjiang 830011 China; The Fifth Affiliated Hospital, Xinjiang Medical University, Urumqi, Xinjiang China; The Municipal Hospital of Aletai, Aletai, Xinjiang China; The Center for Disease Prevention and Control, Tacheng, Xinjiang China

**Keywords:** Apolipoprotein E, Polymorphisms, Primary hyperuricemia, Uygur

## Abstract

**Background:**

Apolipoprotein E (ApoE) participates in lipoprotein metabolism and immune regulation. This study assessed association between *ApoE* polymorphisms with hyperuricemia and uric acid metabolism in Uygur men, Xinjiang, China.

**Methods:**

A total of 474 hyperuricemia patients and 518 healthy male controls were recruited from the Health Screening Center, Uygur region of Xinjiang, China and subjected to ApoE genotyping using a multiplex amplification refractory mutation system PCR.

**Results:**

Apolipoprotein *E3/3* genotype was the predominant type with a frequency of 67.7%, while *E2/2* was lower than *E4/4* in Uygur males. The frequencies of *ApoE2, E3,* and *E4* alleles were 8.5%, 80.1% and 11.4%, respectively. Distribution of ApoE genotypes was significantly different in hyperuricemia patients from the healthy controls (*p <* 0.001). Particularly, the frequency of *ApoE E3/3* was 71.7%, *E2/3* 9.3%, *E3/4* 9.3%, *E4/4* 3.2%, *E2/4* 2.3%, and *E2/2* 0.2% in patients vs. 68.1%, 4.6%, 2.9%, 12%, 0.6%, and 4.6% in controls, respectively. Moreover, frequency of *ApoE* E2 allele was greater in the healthy controls than in patients (*p <* 0.001) and the highest level of uric acid occurred in those with *ApoE2/4* and *E3/4* genotypes, whereas the lowest uric acid level occurred in those with *ApoE E2/2* genotype. In addition, the subjects with the ApoE2 allele had a lower uric acid and LDL-C level than those with the ApoE3 allele and ApoE4 allele (p < 0.05). The risk of developing hyperuricemia in subjects without the ApoE2 allele was 1.7 fold higher than those subjects with the ApoE2 allele.

**Conclusions:**

This study revealed frequencies and distributions of *ApoE* alleles and genotypes in Uygur males, which are different from Han Chinese. *ApoE* E4 was associated with a slightly higher risk of primary hyperuricemia, whereas *ApoE* E2 was associated with reduced risk of primary hyperuricemia and LDL-C level.

## Background

Hyperuricemia refers to an abnormally high serum level of uric acid and commonly occurs in patients with lipoprotein metabolism disorders, metabolic syndrome and cardio-vascular diseases [1]. In China, the incidence of primary hyperuricemia in men has been reported as 21.6% (95% CI, 18.9%-24.6%) [2]. During the past decades, the mean serum uric acid levels and the prevalence of hyperuricemia in the general population appear to be increasing, while the prevalence and incidence of hyperuricemia-induced gout has doubled in the United States of America [3]. Etiologically, many factors can contribute to hyperuricemia, such as genetic susceptibility, insulin resistance, hypertension, renal insufficiency, obesity, diet, and consumption of alcoholic beverages [4]. Among these, alcohol, diet, oxidative stress, and genetic factors play important roles in the pathogenesis of hyperuricemia [5]. However, it remains to be defined which genetic factors contribute to the development of hyperuricemia.

Apolipoprotein E (ApoE) is a multifunctional protein that plays an important role in lipoprotein metabolism [6]. Altered expression or genetic polymorphisms was considered as a risk factor for metabolic syndrome [7] and more recent studies have shown that ApoE also participates in immunoregulation by suppression of T lymphocyte proliferation and regulation of macrophage function [8]. ApoE may also be involved in the development of sporadic Alzheimer disease [9-11] or other diseases [12-16]. Thus, ApoE has become a focus for investigation of its role in metabolic diseases. For example, previous studies showed that in the pathogenesis of hyperuricemia, ApoE polymorphisms in patients with gout were reported to be associated with reduced renal excretion of urates [17,18].

*ApoE* is mapped to chromosome 19 (19q13.2) [19] and contains four exons. *ApoE* polymorphisms affect three common alleles (designated E2, E3, and E4). *ApoE* E3 allele has a cysteine at position 112 and an arginine at 158, while *ApoE* E4 has an arginine at position 112, and *ApoE* E2 has a cysteine at position 158. Although there are just one or two amino acid changes, these ApoE polymorphisms alter ApoE structure and function [20]. In the general population, the prevalence of ApoE2 is between 0.01 and 0.15, ApoE3 between 0.49 and 0.91, and ApoE4 is between 0.06 and 0.37 [15]. ApoE3 most commonly occurs in humans and is found in more than half of the general population [21]. However, distribution of ApoE genotypes differs among populations [6]. Thus, in this study, we aimed to detect the frequency of ApoE polymorphisms in Uygur males and assess how ApoE polymorphisms were associated with primary hyperuricemia risk.

## Results

### Characterization of study population

Summary statistics of characterizations are shown in Table 1. The data showed that body weight and BMI of hyperuricemia patients were smaller than that of healthy controls (*p <* 0.05). Most of the blood tests were abnormal in patients, whereas all were normal in the healthy controls (Table 1).

**Table 1:** **Association of clinical data and apoE genotype between hyperuricemia patients and control group**

**Clinicopathological data**	**Control group (mean ± SD) (** ***n*** **= 518)**	**Hyperuricemia patients (mean ± SD) (** ***n*** **= 474)**	***p*** **-value/**
Age (yrs.)	46.60 ± 11.87	47.28 ± 13.44	0.401
Stature (cm)	169.94 ± 5.11	170.28 ± 5.39	0.312
Weight (kg)	77.38 ± 9.23	80.99 ± 14.19*	<0.05
BMI (kg/m^2^)	26.79 ± 2.96	27.88 ± 4.42*	<0.001
WC (cm)	93.72 ± 8.62	97.51 ± 11.57*	<0.001
Hip circumferences (cm)	101.69 ± 7.15	102.81 ± 10.57*	0.045
WHR	0.92 ± 0.05	0.95 ± 0.07*	<0.001
SBP (mmHg)	110.81 ± 16.34	118.28 ± 18.21*	<0.001
DBP (mmHg)	95.81 ± 21.46	103.86 ± 23.38*	<0.001
BUN (mg/dl)	5.23 ± 1.44	6.47 ± 3.57*	<0.001
Cr (mg/dl)	77.44 ± 18.14	88.91 ± 35.23*	0.003
SUA (mmol/L)	308.51 ± 60.25	495.42 ± 83.66*	<0.001
FBS (mmol/L)	5.18 ± 1.16	5.41 ± 1.80*	0.017
TG (mmol/L)	2.88 ± 1.76	2.75 ± 1.57*	0.005
TC (mmol/L)	4.19 ± 1.48	4.24 ± 1.41	0.594
HDL-C (mmol/L)	1.20 ± 0.43	1.13 ± 0.61*	0.043
LDL-C (mmol/L)	2.65 ± 0.88	2.74 ± 0.77	0.088
Genotype*	N (%)	N (%)	χ^*2*^ *value*
E2/2	24 (4.6)	1 (0.2)	0.000
E2/3	61 (11.8)	44 (9.3)	
E2/4	3 (0.6)	11 (2.3)	
E3/3	353 (68.1)	359 (75.7)	
E3/4	15 (2.9)	44 (9.3)	
E4/4	62 (12.0)	15 (3.2)	
Allele			0.000
E2	112 (10.8)	57 (6.0)	
E3	782 (75.7)	806 (85.0)	
E4	139 (13.5)	85 (9.0)	

A *t*-test and Pearson Chi-square (χ^2^) test were used to analyze the data. *The mean difference was significant at the 0.05 level. Data are presented as range, mean ± standard deviation, or percentage. BMI, Body Mass Index; WC, Waist Circumference; WHR, Waist Hip Ratio; SUA, Serum Uric Acid; TG, Serum Triglyceride; TC, Serum Total Cholesterol; HDL-C, Serum High Density Lipoprotein; LDL-C, Serum Low Density Lipoprotein; FBS, Fasting Blood Sugar; BUN, Urea Nitrogen; SCR, Serum Creatinine.

### Comparison of ApoE allele frequencies and genotype distribution in hyperuricemia with male Uygur controls

**Figure 1:**
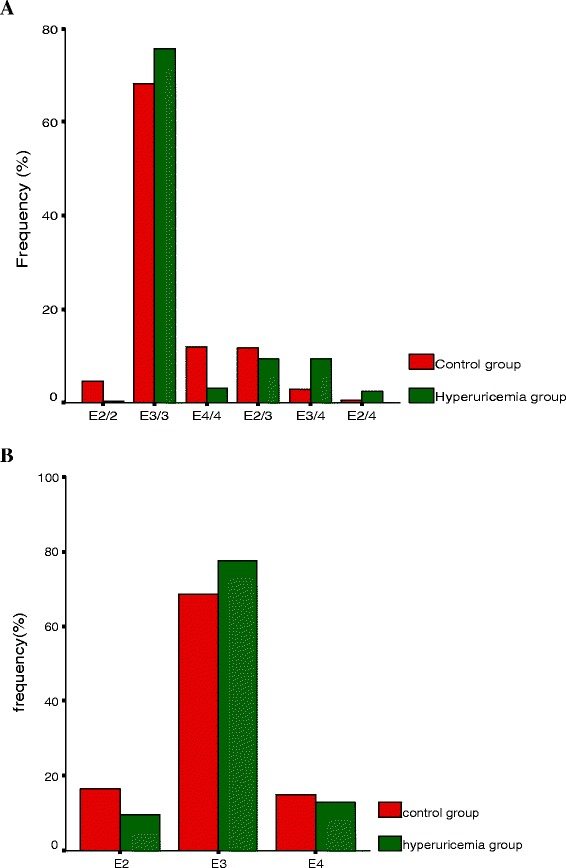
Frequency of ApoE genotypes and alleles in hyperuricemia patients and controls. **A**, Genotypes and **B**, Alleles.

The distribution of *ApoE* genotypes was consistent with the Hardy-Weinberg equilibrium. The frequencies of *ApoE2*, *E3* and *E4* were approximately 8.5%, 80.05%, and 11.45% in these studied subjects, respectively. Table 1 and Figure 1A showed the distribution of *ApoE* genotypes differed between hyperuricemia and control subjects in Uygur men (χ^2^ = *69.662*, *P* = 0.000). The most common genotype in the hyperuricemia group was *E3/3* (71.7%), followed by *E2/3* (9.3%), *E3/4* (9.3%), *E4/4* (3.2%), *E2/4* (2.3%), and *E2/2* (0.2%), whereas those in control subjects were *E3/3* (68.1%), followed by *E4/4* (12%), *E2/3* (11.8%), *E2/2* (4.6%), *E3/4* (2.9%), and *E2/4* (0.6%). Furthermore, Table 1 and Figure 1B showed the distribution of *ApoE* alleles was different between hyperuricemia and control (χ^2^ = 27.684, ***P*** = 0.000). Thus, the frequency of the *ApoE2* allele in control subjects (10.8%) was greater than in the hyperuricemic patients (6%; *p* < 0.05).

### Comparison of uric acid level and hyperuricemia incidence among the different ApoE genotypes and alleles

**Figure 2:**
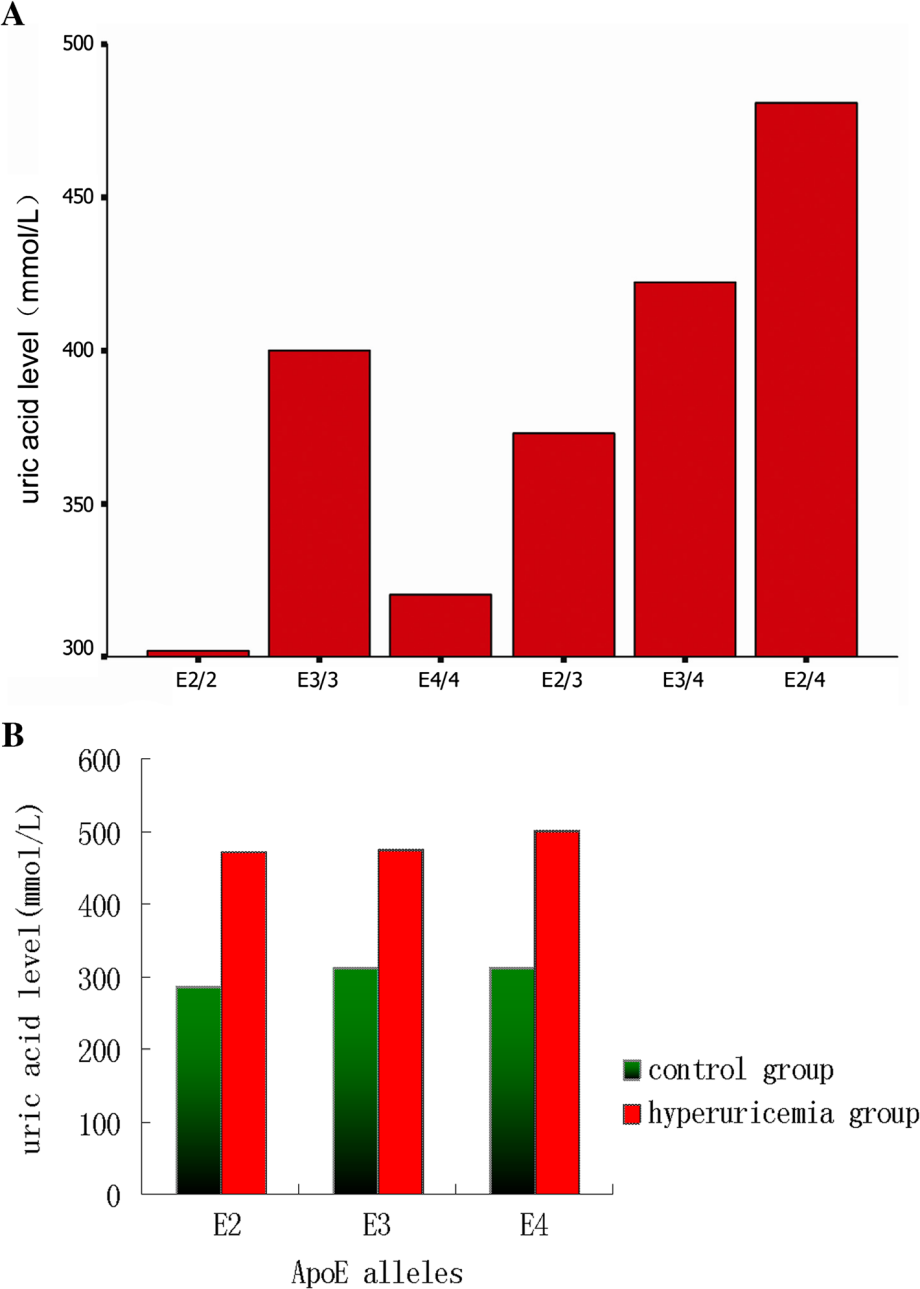
Association of ApoE genotypes and alleles with serum level of uric acid. **A**, Genotypes and **B**, Alleles.

**Figure 3:**
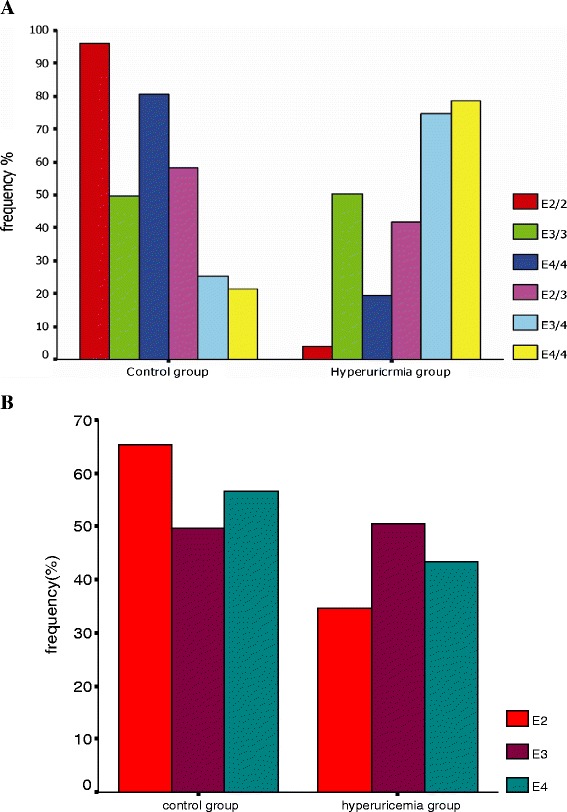
Association of ApoE genotypes and alleles with risk of hyperuricemia. **A**, Genotypes and **B**, Alleles.

These ApoE genotypes were associated with the highest level of uric acid and hyperuricemia incidence (*p* < 0.001). Specifically, the subjects with an ApoE2/4 genotype had a higher level of serum uric acid and risk in developing hyperuricemia, followed by *ApoE3/4, E3/3, E2/3*, and *E4/4.* However, subjects with the *ApoE2/2* genotype had the lowest level of serum uric acid level and a lower hyperuricemia incidence (Table 2, Figure 1A, Figure 2A, and Figure 3A).

**Table 2:** **Association of ApoE genotypes with serum uric acid level and hyperuricemia patients and controls**

**Genotypes**	**N**	**SUA level**	**Hyperuricemia patients**	**Controls**
		Mean ± SD	N (%)	N (%)
E2/2	25	318.52 ± 72.29	1(4.0)	24(96.0)
E3/3	712	407.32 ± 119.78	359(50.4)	353(49.6)
E4/4	77	324.74 ± 83.67	15(19.5)	62(80.5)
E2/3	105	380.85 ± 104.21	44(41.9)	61(58.1)
E3/4	59	425.24 ± 110.52	44(74.6)	15(25.4
E2/4	14	469.87 ± 165.93	11(78.6)	3(21.4)
Total	992	397.82 ± 118.15	474(47.8)	518(52.2)
F value/χ^2^		11.784	69.662	
p value		<0.001	< 0.001	

**One-way analysis of variance (ANOVA) was used to analyze the differences.

**Pearson Chi-square (χ^2^) test was used to analyze the differences. SUA, serum uric acid.

However, the subjects without the *ApoE2* allele had a higher uric acid level than those with the *ApoE2* allele (*p* <0.05; Table 2, Figure 2B, and Figure 3B). Likewise, the incidence of hyperuricemia was also higher in subjects without the *ApoE2* allele (*p <* 0.001) and the risk of developing hyperuricemia was 1.7 fold higher than those subjects with the *ApoE2* allele. Moreover, compared to the subjects with the *ApoE4* allele*,* serum uric acid levels in the subjects without the *ApoE4* allele was higher (*p <* 0.05; Table 3), but did not augment the risk of developing hyperuricemia (*p >* 0.05).

**Table 3:** **Association of the mean SUA level and hyperuricemia incidence between the subjects with and without** ***ApoE2*** **and** ***E4*** **allele**

	***E*** **2 allele(n = 130)**	**w/o** ***E*** **2 allele(n = 862)**	***P*** **value**	***OR***
SUA level (Mean ± SD)	368.86 ± 101.6	402.19 ± 119.8	0.003	
Hyperuricemia (%)	34.6	49.8	0.000	1.728
	*E*4 allele(n = 147)	w/o *E*4 allele(n = 845)	*P* value	*OR*
SUA level (Mean ± SD)	379.35 ± 118.2	401.03 ± 117.9	0.040	
Hyperuricemia (%)	47.6	47.8	0.966	1.007

Pearson’s chi-square test was used to analyze the data. *The mean difference was significant at the 0.05 level. w/o, Without. OR, Relative risk.

Moreover, we also compared other biochemical parameters with different ApoE genotypes between hyperuricemia patients and controls and found that both groups of subjects with the *ApoE2* allele had a lower uric acid and LDL-C level than those with the *ApoE3* allele and *ApoE4* allele (*p <*0.05; Table 4).

**Table 4:** **Comparison of the biochemical parameters between different ApoE alleles in hyperuricemia patients and controls**

**Groups**	**Variable(s)**	***ApoE2*** **(85)**	***ApoE3*** **(353)**	***ApoE4*** **(77)**	**Total (478)**	***p*** ^***a***^	***p*** ^***b***^	***p*** ^***c***^
control group	BUN (mg/dl)	5.15 ± 1.42	5.23 ± 1.46	5.30 ± 1.44	5.22 ± 1.45	0.675	0.682	0.516
	Cr (mg/dl)	75.54 ± 15.60	76.99 ± 18.88	80.53 ± 16.74	77.36 ± 18.12	0.523	0.107	0.080
	SUA (mmol/L)	287.93 ± 57.89	312.24 ± 59.89	311.86 ± 61.03	308.54 ± 60.30	0.001*	0.958	0.011*
	FBS(mmol/L)	5.29 ± 1.35	5.19 ± 1.18	5.03 ± 0.82	5.18 ± 1.16	0.480	0.260	0.149
	TG(mmol/L)	2.13 ± 1.52	2.58 ± 1.8	2.29 ± 1.59	2.46 ± 1.77	0.037*	0.188	0.578
	TC(mmol/L)	4.17 ± 1.38	4.20 ± 1.50	4.15 ± 1.54	4.19 ± 1.48	0.863	0.791	0.936
	HDL-C(mmol/L)	1.14 ± 0.44	1.22 ± 0.45	1.20 ± 0.34	1.20 ± 0.44	0.158	0.825	0.364
	LDL-C(mmol/L)	2.45 ± 0.63	2.81 ± 0.82	2.78 ± 0.67	2.75 ± 0.78	0.000*	0.710	0.008*
Groups	Variable(s)	*ApoE2 (45)*	*ApoE3 (359)*	*ApoE4 (59)*	*Total (463)*	*p* ^*a*^	*p* ^*b*^	*p* ^*c*^
	BUN (mg/dl)	5.74 ± 2.29	6.51 ± 3.44	6.20 ± 2.16	6.39 ± 3.21	0.135	0.503	0.470
	Cr (mg/dl)	79.64 ± 30.99	87.13 ± 25.21	90.17 ± 37.16	88.76 ± 35.37	0.060	0.540	0.285
Hyperuricemia patients	SUA (mmol/L)	473.29 ± 55.02	476.53 ± 71.12	500.82 ± 85.37	494.95 ± 81.42	0.840	0.058*	0.016*
	FBS(mmol/L)	5.38 ± 2.07	5.39 ± 1.69	5.39 ± 2.17	5.39 ± 1.79	0.938	0.999	0.950
	TG(mmol/L)	2.44 ± 1.13	2.77 ± 1.0	2.71 ± 1.4259	2.73 ± 1.53	0.168	0.775	0.369
	TC(mmol/L)	4.29 ± 1.54	4.16 ± 1.39	4.54 ± 1.21	4.22 ± 1.39	0.532	0.047*	0.363
	HDL-C(mmol/L)	1.23 ± 0.87	1.12 ± 0.55	1.14 ± 0.64	1.13 ± 0.60	0.221	0.810	0.419
	LDL-C(mmol/L)	2.33 ± 1.00	2.71 ± 0.82	2.72 ± 1.00	2.67 ± 0.87	0.006*	0.929	0.023*

A *t*-test was used to analyze the data. *The mean difference was significant at the 0.05 level. *p* ^a^, *p* values that were obtained when comparing *ApoE2* subjects with *ApoE3* subjects. *p* ^b^, *p* values that were obtained when comparing subjects with *ApoE3* subjects. *ApoE4. p* ^c^, *p* values that were obtained when comparing subjects with *ApoE2* subjects. *ApoE4.*

(1) *ApoE3* group (subjects carrying the E3/3 genotype), (2) *ApoE2* group (subjects carrying the E2/2 or E2/3 genotype), (3) *ApoE4* group (subjects carrying the E4/4 or E3/4 genotype). Subjects with the E2/4 genotype (n = 14) were excluded from the extra analyses because of the opposite effects of the E2 and E4 alleles on the lipid levels.

## Discussion

The distribution of *ApoE* genotypes and alleles varies among different races and geographic areas [22]. In general, the frequency of ApoE E3/3 is higher (>60%) than that of other genotypes, whereas *ApoE4/4*, *E2/4* and *E2/2* have the lowest frequency. In China, the frequency of the ApoE3 allele is approximately 85.2%, which is higher than that of the Caucasian population (76.9%), whereas the ApoE4 allele is about 6.4%, which is obviously lower than that of the Caucasian population (14.3%) [23]. In the current study, we found that the distribution of the ApoE3 allele in Uygur males was over 80%, which is between that found in the Chinese and Caucasian populations. Our data may suggest the Uygur as a separate ethnic group in its own right. Indeed, a previous study of the origin of Human species utilized DNA sequencing technology to assess the Uygur mummy unearthed 3,000 years ago from a dry desert in the Tarim region of Xinjiang, China and showed that the Xinjiang Uygur ethnic originated from Europe [24,25]. Our current data on the *ApoE* polymorphism may provide additional evidence. However, after approximately 3000 years of evolution, one may expect that the ApoE3 allele may be also changed.

ApoE protein is a major component in the very-low-density lipoprotein (VLDL) and the latter helps to remove excess cholesterol from the blood and carry it to the liver for metabolism. ApoE protein also helps to clearance chylomicrons and VLDL from the bloodstream and acts as a ligand to bind to the hepatic receptors, including the low-density lipoprotein (LDL) receptor, LDL receptor–related protein, and VLDL receptor. ApoE2 and E4 are functionally and metabolically different from the common form of ApoE3. For example, ApoE2 has poor affinity to the hepatic lipoprotein receptor, resulting in decreased catabolism of triglyceride-rich lipoproteins and accumulation of VLDL and chylomicron remnants in the blood [13], whereas ApoE4 is associated with the increased clearance of these lipoproteins, resulting in hypercholesterolemia and elevated levels of LDL [6]. In the current study, we also compared other metabolic indicators that connected lipid metabolism with renal functions between different ApoE genotypes in hyperuricemia patients and controls and found both groups of subjects with the *ApoE2* allele had a lower LDL-C level than those with the *ApoE3* allele and *ApoE4* allele, while the subjects with the *ApoE4* allele had the highest LDL-C level than other groups, which was consistent with other studies [6].

The possible association of ApoE and risk of hyperuricemia has been widely investigated in different populations [21]. However, to date, there is no study on the under-represented Uygur in Xinjiang. In the current study, we aimed to understand the possible association of ApoE with risk of hyperuricemia in Uygur population. Our data showed that although ApoE 3/3 genotype is the most common genotype in Uygur, the frequency of E2/2 genotype is lower than the E4/4 genotype in both control and hyperuricemia subjects. The frequency of The frequency of The frequency of indicating that ApoE2 may have a protective function for primary hyperuricemia.

Previous studies of different populations showed that the incidence of primary hyperuricemia has increased significantly in recent years and that the onset tends to occur at younger age [1]. Clinically, hyperuricemia is closely related to obesity, hyperlipidemia, hypertension, diabetes, atherosclerosis and other diseases [26]. It has been noted that the ApoE gene polymorphism plays a role in the development of gout, primary hyperuricemia, and hypertriglyceridemia. For example, Moriwaki et al. [27] reported that the frequency of ApoE4 was higher in patients with gout and hypertriglyceridemia than those with hypertriglyceridemia alone. Furthermore, besides being related to obesity and alcoholism, ApoE4 can also increase incidence of hypertriglyceridemia in patients with gout, thus being a risk factor for atherosclerosis in gout patients. Cardona et al. [17] investigated the correlation between ApoE allele and renal excretion of urate in 68 gout patients and 50 normal controls and found that in gout patients with ApoE2, the levels of TG and VLDL were significantly higher but the renal excretion of urates was decreased. They concluded that the reduction of renal excretion of urates was mediated by the high level of VLDL and ApoE2. Uygur ethnics are a special population in Xinjiang, China, which live in a dry and hot area; thus, they have different lifestyles and food consumption from other provinces in China. Their diet contains high-fat, high protein, and sugar, but little fish and vegetables. Obesity in the urban Uygar population is high. All of these may contribute to primary hyperuricemia, hypertension, diabetes, and other metabolic diseases. Our current study showed that frequencies of the ApoE genotype and alleles were unique in this population and associated with hyperuricemia risk. Our current data are consistent with previous studies by Moriwaki [27], Elmadbouh et al. [28] and others [29]. Furthermore, our current study also showed that the SUA level in the male Uygur was statistically significant between different genotypes i.e. subjects with ApoE2/4 and E3/4 genotype were associated with a higher SUA level and hyperuricemia risk, which is also consistent with the data reported by Cardona et al. [7,23]. It has been noted that the *ApoE* polymorphism was associated with development of gout and primary hyperuricemia. However, Moriwaki et al. [30] analyzed 221 male gout patients and 141 male controls and found that there was no statistically significant difference in frequencies of ApoE allele (E2, E3, or E4) between gout patients and hyperuricemia, and the healthy controls.

## Conclusions

The distribution of ApoE alleles and genotype frequencies in the Uygur male is unique, and was associated with hyperuricemia risk. *ApoE* E4 was associated with a slight increased risk of primary hyperuricemia, whereas *ApoE* E2 was associated with protection against primary hyperuricemia and LDL-C in Uygur men in China. However, our current study is just proof-of-principle and much more data are needed to confirm the link of ApoE polymorphisms with the risk of primary hyperuricemia.

## Methods

### Participants and study design

This case–control study recruited 992 subjects selected from the Affiliated Hospitals of Xinjiang Medical University (Uygur, China). They were all from Uygur and the cases contained 474 patients with hyperuricemia, confirmed by laboratory tests (serum uric acid levels were more than 417 μM), whereas the gender and age-matched healthy control subjects had no history of hyperuricemia. This study was approved by the Ethics Committee of the First Affiliated Hospital of Xinjiang Medical University and was conducted according to the standards of the Declaration of Helsinki. All participants were fully informed of the purpose of this study and all subjects provided written informed consent before enrollment into the study.

### Inclusion and exclusion criteria

We recruited Uygur men aged between 18 to 70 years as the hyperuricemia group, who had serum uric acid levels more than 417 μmol within 2 weeks before entering this study. The control group was matched with the hyperuricemia group for age and gender. The exclusion criteria were i) acute onset of gouty arthritis or renal stone; ii) significant liver or renal dysfunction, hematological disease, oncological disease, or other life threatening disorders; iii) conditions that need the management of diuretics or analgesic agents; and iv) receiving any anti-hyperuricemia agents.

### Date collection and blood tests

A questionnaire was used to collected data on the demographic, lifestyle, and disease history from all participants. Physical examination was also performed on all participants, including height (measured in centimeter for an error less than 0.5 cm), bodyweight (measured in kilogram for an error less than 0.1 kg), body mass index (BMI), waist circumference (WC), and hip circumference (calibrated weekly to within 1 mm using plastic tapes), blood pressure and blood tests (see below). The waist circumference was measured at the end of a gentle expiration midway between the lowest rib and iliac crest, with the study participant standing, while the hip circumference was measured at the greater trochanter. The waist hip ratio (WHR) was determined as waist (cm) divided by hip (cm). Blood pressure was measured using the automatic clinical blood pressure monitor three times in the sitting position following a standard protocol. All participants were at rest at least 10 min before the physical examination.

All participants were also asked to fast for at least 12 h and not to consume any alcohol or high-fat foods on the previous night before blood withdrawal the next morning. Each subject had two mL of venous blood withdrawn to assess serum uric acid (SUA), triglyceride (TG), total cholesterol (TC), high-density lipoprotein (HDL-C), low-density lipoprotein (LDL-C), fasting blood sugar (FBS), urea nitrogen (BUN), and creatinine (SCR), measured by the 7060 Automatic Biochemical Analyzer (Hitachi, Ltd., Tokyo, Japan).

### Genomic DNA extraction and ApoE genotyping

Genomic DNA was extracted from blood cells by the salting-out method [31,32] and subjected to ApoE genotyping using Gerard’s method with modifications in the multiplex amplification refractory mutation system PCR (Multi-ARMS PCR) [30,33]. The primers were designed and synthesized by the Biological Products Engineering Co., Ltd. (Shanghai, China) for ApoE (5'-GTTCAGTGATTGTCGCTGGGCA-3' that paired with Arg/Cyys158/Arg158 (5'-ATGCCGATGACCTGCAGAATT-3' or 5'-ATGCCGATGACCTGCAGAATC-3'), Cys112/Arg112 (5'-CGCGGACATGGAGGACGTTT-3' or 5'-CGCGGACATGGAGGACGTTC-3'). A common primer was ds 158 or Arg/Cys 112 and produced an amplicon of 588 bp and 451 bp, respectively. The PCR mixture in 25 μL contained 50 ng of genomic DNA, 200 μmol/L of dNTPs (Biological Products Engineering Co., Ltd), PCR buffer with 10 mmol/L Tris–HCl, pH 8.8, 1.5 mmol/L MgCl_2_, 50 mmol/L KCl, and 1 mL/L Triton X-100 from Finnzymes (Shanghai, China), 80 g/L dimethyl sulfoxide (DMSO; Sigma), 1 U of DyNAzyme II DNA Polymerase (Finnzymes), 8 nmol/L of α1-antitrypsin primers, and 0.8 μmol/L ARMS common primer. The reaction mixture A in addition to the above also contained 0.8 μmol/L Cys158 and 0.4 μmol/L Cys112 primers. Similarly, the reaction mixture B contained 0.8 μmol/L Arg158 and 0.4 μmol/L Arg112 primers. The PCR amplification was set to an initial denaturation at 95°C for 4 min and then 35 cycles of 96°C for 45 s, 66°C for 45 s, and 72°C for 45 s, and followed by a final extension at 72°C for 5 min. The PCR product (9 μL) was mixed with 3 μL of 6 × gel loading dye type I (Sigma) and separated on a 1.6% agarose gel (Sigma) that contained 0.1 mg/L ethidium bromide (Bio-Rad, Hercules, CA, USA).

### Statistical analysis

The data are summarized as numbers, percentage, or mean ± standard deviation and then organized in Epidata 3.0 software (The EpiData Association, Odense, Denmark) and analyzed by using SPSS 16.0 for windows software package (SPSS, Chicago, IL, USA). Simple descriptive statistics were used to describe the variables among the participants. The differences of measurements from different groups were compared with student *t*-test and One-way analysis of variance (ANOVA). ApoE genotypes and frequencies were analyzed with Pearson’s chi-square test, if genotypes met the Hardy-Weinberg equilibrium. A *p* value < 0.05 was considered to be statistically significant.

## Abbreviations

ApoE: Apolipoprotein E
LDL: Low-density lipoprotein
BMI: Body mass index
WC: Waist circumference
WHR: Waist hip ratio
SUA: Serum uric acid
TG: Triglyceride
TC: Total cholesterol
HDL-C: High-density lipoprotein
LDL-C: Low-density lipoprotein
FBS: Fasting blood sugar
BUN: Urea nitrogen
SCR: Creatinine
